# Supplementary material for: Comparative Genomic Analysis of the Poaceae Cytokinin Response Regulator RRB Gene Family and Functional Characterization of OsRRB5 in Drought Stress Tolerance in Rice
Source: Int J Mol Sci. 2025 Feb 24;26(5):1954. doi: 10.3390/ijms26051954 (PMC11899991; doi:10.3390/ijms26051954)
Supplement: Supplementary file 1 [file ijms-26-01954-s001.zip › ijms-3432360-supplementary.pdf]

Supplementary information for

Comparative genomic analysis of the Poaceae cytokinin response regulator *RRB* gene family and functional characterization of *OsRRB5* in drought stress tolerance in rice

Rujia Chen[1,2,3,#], Qianfeng Huang[1,2,#], Yanan Xu[1,2], Zhichao Wang[1,2], Nian Li[1,2], Yue Lu[1,2], Tianyun Tao[1,2], Yu Hua[1,2], Gaobo Wang[1,2], Shuting Wang[1,2], Hanyao Wang[1,2], Yong Zhou[1,2], Yang Xu[1,2], Pengcheng Li[1,2], Chenwu Xu[1,2,3,*], Zefeng Yang[1,2,3,*]

[1] Jiangsu Key Laboratory of Crop Genomics and Molecular Breeding/Zhongshan Biological Breeding Laboratory/Key Laboratory of Plant Functional Genomics of the Ministry of Education/Jiangsu Key Laboratory of Crop Genetics and Physiology, Agricultural College of Yangzhou University, Yangzhou 225009, China

[2] Jiangsu Co-Innovation Center for Modern Production Technology of Grain Crops, Yangzhou University, Yangzhou 225009, China

[3] Joint International Research Laboratory of Agriculture and Agri-Product Safety of the Ministry of Education, Yangzhou University, Yangzhou, 225009, China.

[*] Correspondence: Zefeng Yang, zfyang@yzu.edu.cn, 0514-87979358

Chenwu Xu, cwxu@yzu.edu.cn, 0514-87979358

[#] These authors contributed equally to this work.

This Word file includes:

Figures S1 to S3

Tables S1 to S8

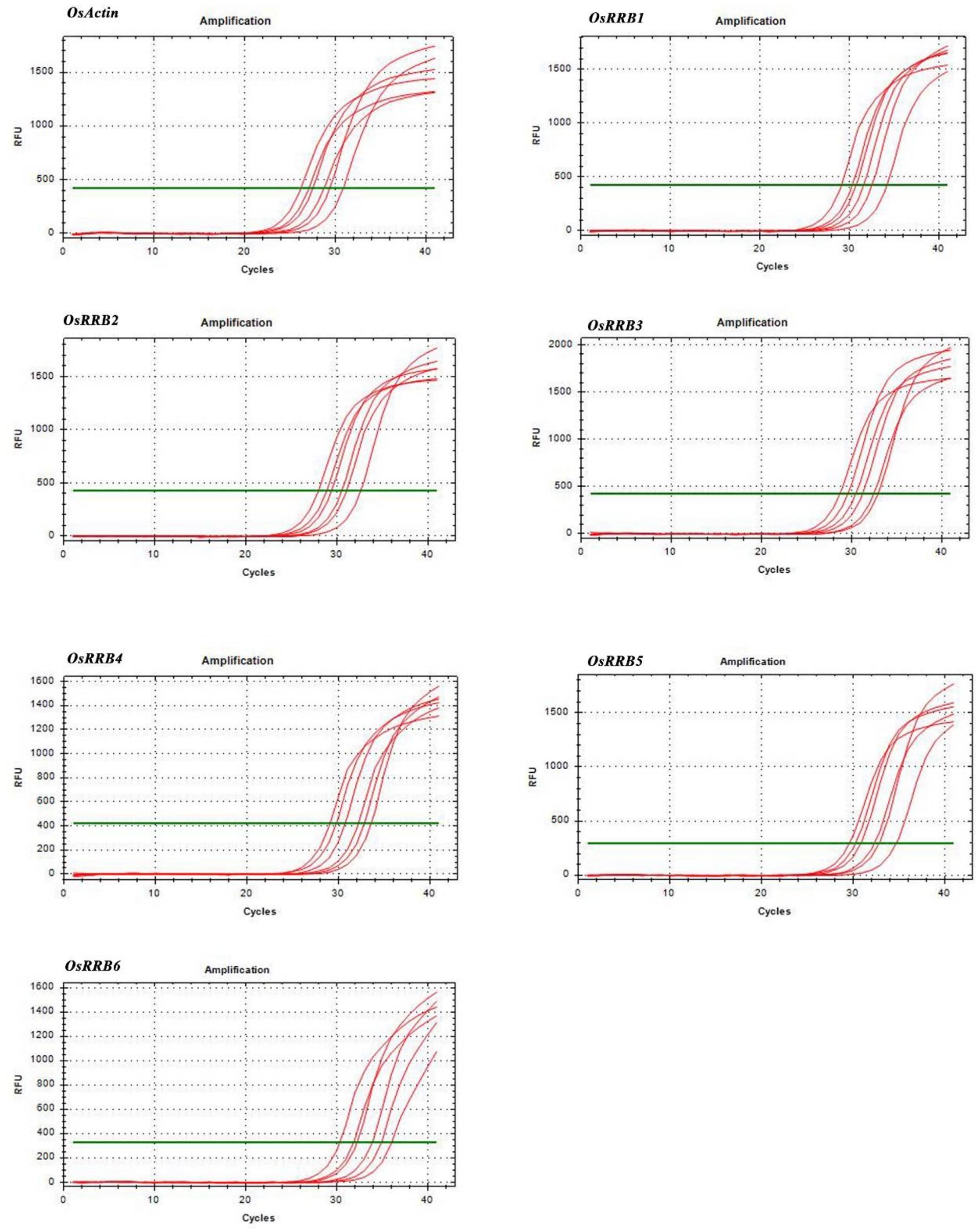

Figure S1: Amplification curves of *OsRRB* and *OsActin* genes in qRT-PCR analysis. The term "RFU" refers to the relative Fluorescence Units. The cDNA concentration was diluted in a series of 1:1, 1:2, 1:4, 1:8, 1:16, and 1:32 dilutions.

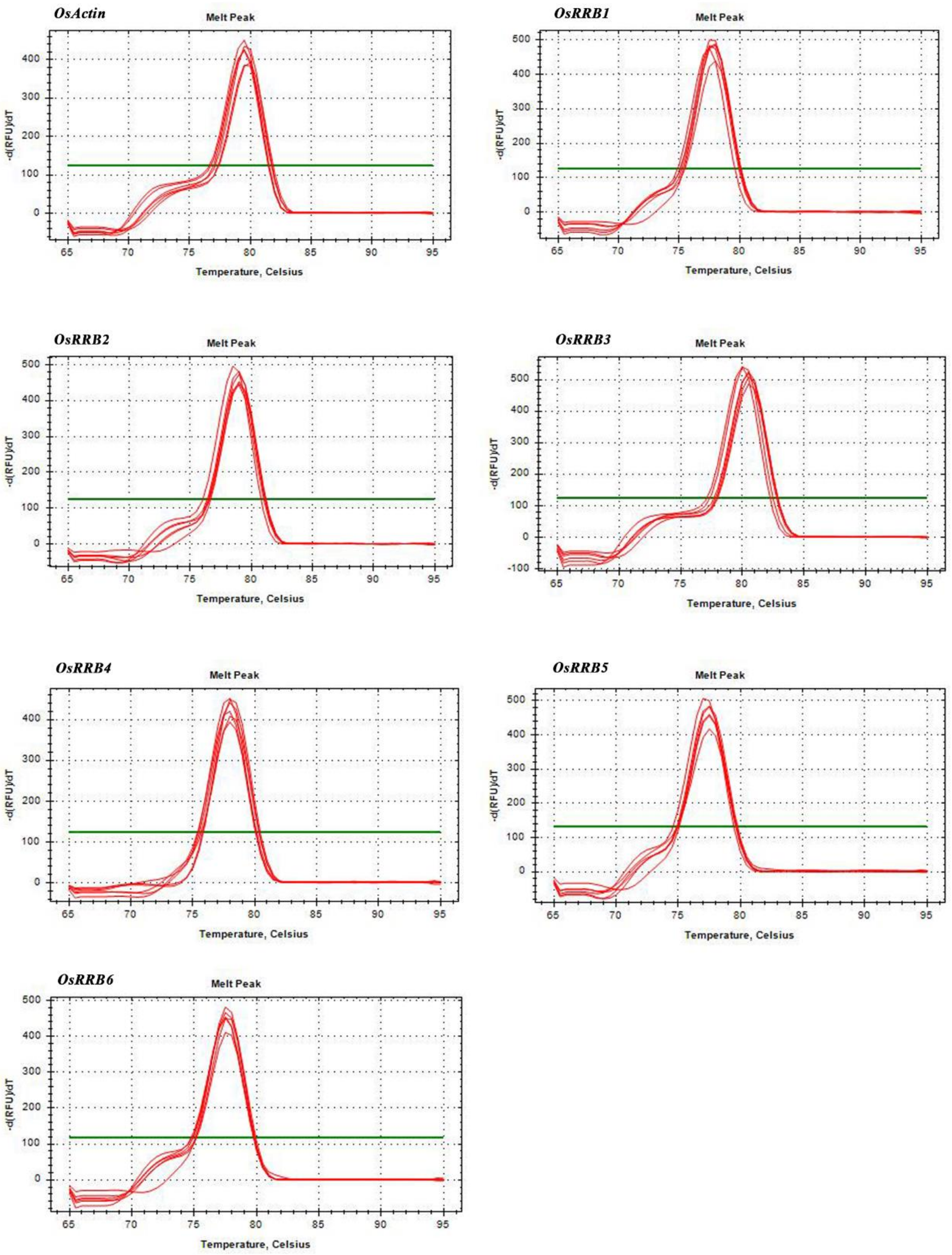

Figure S2: Melting curves of *OsRRB* and *OsActin* genes in qRT-PCR analysis. The cDNA concentration was diluted in a series of 1:1, 1:2, 1:4, 1:8, 1:16, and 1:32 dilutions.

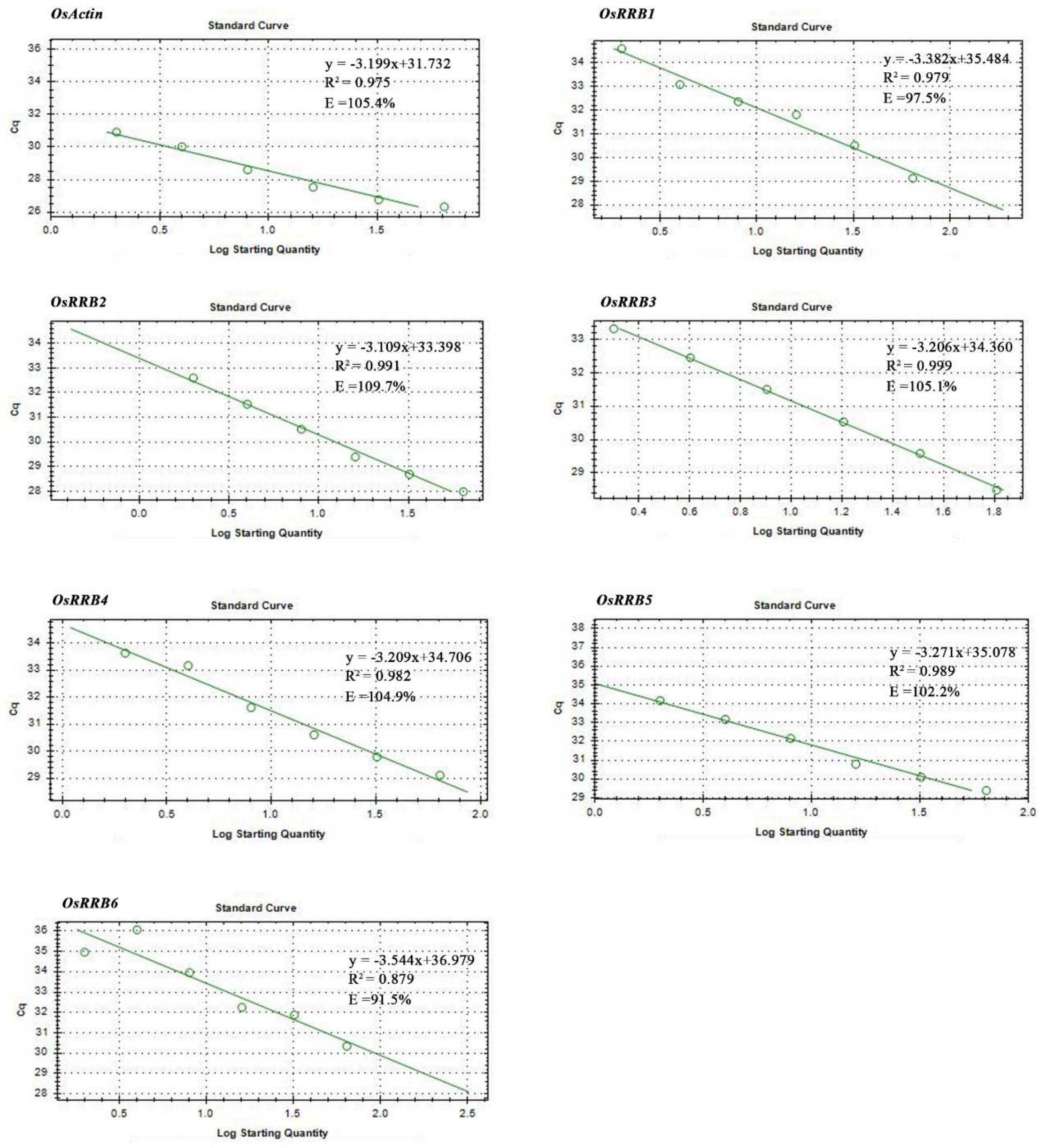

Figure S3. Standard curves and primer efficiency of *OsRRB* and *OsActin* genes in qRT-PCR analysis. The PCR efficiency (E; %) for each primer pair was calculated as $E = (10-1/A - 1) \times 100\%$. The cDNA concentration was diluted in a series of 1:1, 1:2, 1:4, 1:8, 1:16, and 1:32 dilutions. The term "Cq" refers to the quantification cycle value.

Table S1. Information of rice accessions used in Haplotype analysis.

Accession ID
B269
CX220
IRIS_313-10065
IRIS_313-10056
IRIS_313-10059
IRIS_313-10074
IRIS_313-10089
IRIS_313-10082
IRIS_313-10078
IRIS_313-10084
IRIS_313-10097
IRIS_313-10242
IRIS_313-10327
IRIS_313-10379
IRIS_313-10429
IRIS_313-10437
IRIS_313-10558
IRIS_313-10552
IRIS_313-10564
IRIS_313-10563
IRIS_313-10567
IRIS_313-10569
IRIS_313-10568
IRIS_313-10583
IRIS_313-10582
IRIS_313-10620
IRIS_313-10619
IRIS_313-10631
IRIS_313-10642
IRIS_313-10644
IRIS_313-10660
IRIS_313-10677
IRIS_313-10704
IRIS_313-10708
IRIS_313-10710
IRIS_313-10743
IRIS_313-10828
IRIS_313-10830
IRIS_313-10827
IRIS_313-10832
IRIS_313-10829
IRIS_313-10840

IRIS_313-10862
IRIS_313-10865
IRIS_313-10866
IRIS_313-10867
IRIS_313-10884
IRIS_313-10888
IRIS_313-10893
IRIS_313-10895
IRIS_313-10923
IRIS_313-10946
IRIS_313-10949
IRIS_313-10953
IRIS_313-10967
IRIS_313-11003
IRIS_313-10999
IRIS_313-11001
IRIS_313-11004
IRIS_313-11009
IRIS_313-11077
IRIS_313-11075
IRIS_313-11093
IRIS_313-11117
IRIS_313-11155
IRIS_313-11202
IRIS_313-11379
IRIS_313-11396
IRIS_313-11427
IRIS_313-11424
IRIS_313-11436
IRIS_313-11464
IRIS_313-11511
IRIS_313-11495
IRIS_313-11539
IRIS_313-11582
IRIS_313-11660
IRIS_313-11672
IRIS_313-11673
IRIS_313-11900
IRIS_313-11890
IRIS_313-11907
IRIS_313-11913
IRIS_313-11923
IRIS_313-11929
IRIS_313-11928

IRIS_313-11924
IRIS_313-11922
IRIS_313-11926
IRIS_313-12051
IRIS_313-12061
IRIS_313-12059
IRIS_313-12071
IRIS_313-12069
IRIS_313-12073
IRIS_313-12077
IRIS_313-12164
IRIS_313-12200
IRIS_313-12217
IRIS_313-12228
IRIS_313-12242
IRIS_313-12252
IRIS_313-12254
IRIS_313-12244
IRIS_313-12262
IRIS_313-12257
IRIS_313-12265
IRIS_313-12266
IRIS_313-12271
IRIS_313-12281
IRIS_313-12311
IRIS_313-12312
IRIS_313-12324
IRIS_313-12330
IRIS_313-12348
IRIS_313-12345
IRIS_313-12342
IRIS_313-12337
IRIS_313-12332
IRIS_313-12349
IRIS_313-12350
IRIS_313-12346
IRIS_313-15907
IRIS_313-12352
IRIS_313-15905
IRIS_313-7902
IRIS_313-7868
IRIS_313-7907
IRIS_313-7959
IRIS_313-7866

IRIS_313-7870
IRIS_313-7909
IRIS_313-7992
IRIS_313-8011
IRIS_313-7912
IRIS_313-8010
IRIS_313-8023
IRIS_313-7922
IRIS_313-8032
IRIS_313-8031
IRIS_313-8025
IRIS_313-8041
IRIS_313-8027
IRIS_313-8033
IRIS_313-8048
IRIS_313-8044
IRIS_313-8039
IRIS_313-8053
IRIS_313-8049
IRIS_313-8046
IRIS_313-8062
IRIS_313-8052
IRIS_313-8074
IRIS_313-8087
IRIS_313-8095
IRIS_313-8075
IRIS_313-8090
IRIS_313-8096
IRIS_313-8102
IRIS_313-8114
IRIS_313-8097
IRIS_313-8113
IRIS_313-8105
IRIS_313-8115
IRIS_313-8118
IRIS_313-8121
IRIS_313-8126
IRIS_313-8124
IRIS_313-8119
IRIS_313-8116
IRIS_313-8123
IRIS_313-8127
IRIS_313-8132
IRIS_313-8140

IRIS_313-8129
IRIS_313-8135
IRIS_313-8138
IRIS_313-8145
IRIS_313-8136
IRIS_313-8142
IRIS_313-8141
IRIS_313-8139
IRIS_313-8134
IRIS_313-8151
IRIS_313-8155
IRIS_313-8158
IRIS_313-8159
IRIS_313-8170
IRIS_313-8173
IRIS_313-8183
IRIS_313-8186
IRIS_313-8204
IRIS_313-8208
IRIS_313-8205
IRIS_313-8200
IRIS_313-8195
IRIS_313-8214
IRIS_313-8209
IRIS_313-8213
IRIS_313-8356
IRIS_313-8387
IRIS_313-8481
IRIS_313-8486
IRIS_313-8523
IRIS_313-8565
IRIS_313-8578
IRIS_313-8580
IRIS_313-8599
IRIS_313-8658
IRIS_313-8637
IRIS_313-8694
IRIS_313-8755
IRIS_313-8768
IRIS_313-8815
IRIS_313-8865
IRIS_313-8857
IRIS_313-8884
IRIS_313-8890

IRIS_313-9050
IRIS_313-9081
IRIS_313-9193
IRIS_313-9346
IRIS_313-9379
IRIS_313-9389
IRIS_313-9438
IRIS_313-9452
IRIS_313-9539
IRIS_313-9529
IRIS_313-9616
IRIS_313-9698
IRIS_313-9701
IRIS_313-9742
IRIS_313-9724
IRIS_313-9745
IRIS_313-9774
IRIS_313-9782
IRIS_313-9789
IRIS_313-9790
IRIS_313-9813
IRIS_313-9838
IRIS_313-9887
IRIS_313-9884
IRIS_313-9929
IRIS_313-9964
IRIS_313-9974

Table S2. The specific primers for the rice *OsRRB* genes used in qRT-PCR analysis.

**Primer name**	**Sequence (5'-3')**
OsRRB1-F	TGTTCCTACGAAGTCACCACA
OsRRB1-R	CCAGAAGCTTGAAACCATCCA
OsRRB2-F	ACTGCGGTTCCATCCAACAT
OsRRB2-R	GCATCTCATTTCCCAGCAGC
OsRRB3-F	GGAGATATGGCGATGCCGAA
OsRRB3-R	GGTCGTCGCTGAAGGTAACA
OsRRB4-F	TGGTATGGCAGACTCAACCG
OsRRB4-R	AAGGGTTCCTGACAGCATGG
OsRRB5-F	GCATGCTTGTCTCCCCTTCT
OsRRB5-R	AGAATTGCTTGCACCTCCGA
OsRRB6-F	GACACAAGCCCAGCAGAGAT
OsRRB6-R	TCTCAGGGCTGTCGAGATGA
OsActin-F	CCCCTCCTGAAAGGAAGTA
OsActin-R	GGTCCGAAGAATTAGAAGCA

Table S3. The quantification cycle (Cq) values of *OsRRB1* and *OsActin* in qRT-PCR analysis.

Treatments		*OsActin*			*OsRRB1*	
**6-BA-0h**	23.14	23.01	23.03	27.08	27.05	27.13
**6-BA-1h**	23.12	23.00	22.98	27.92	27.82	27.84
**6-BA-3h**	20.89	20.77	20.77	27.48	27.44	27.44
**6-BA-6h**	21.68	21.31	21.20	27.48	27.44	27.46
**6-BA-9h**	21.76	21.59	21.53	27.54	27.77	27.64
**6-BA-12h**	22.40	22.08	22.10	28.15	28.20	28.08
**MeJA-0h**	26.50	26.33	26.25	32.06	32.23	32.31
**MeJA-1h**	26.09	26.15	26.26	31.60	31.75	31.40
**MeJA-3h**	26.18	26.18	26.16	33.40	33.73	33.33
**MeJA-6h**	26.16	26.25	26.21	33.88	33.83	33.82
**MeJA-9h**	25.10	25.29	25.23	31.12	31.50	31.38
**MeJA-12h**	25.38	25.45	25.50	32.06	32.02	31.91
**ABA-0h**	24.00	23.98	23.81	32.07	32.48	32.23
**ABA-1h**	25.87	25.69	25.91	33.07	33.02	33.11
**ABA-3h**	26.04	26.09	26.14	34.96	34.36	34.79
**ABA-6h**	26.68	26.59	26.57	34.36	34.08	34.59
**ABA-9h**	26.91	26.99	26.69	37.69	37.63	37.74
**ABA-12h**	25.61	25.47	25.33	35.21	35.05	35.19
**Cold-0h**	25.76	25.84	25.68	32.81	32.74	32.39
**Cold-1h**	25.42	25.42	25.29	32.65	32.57	32.45
**Cold-3h**	25.70	25.63	25.43	31.78	31.78	31.92
**Cold-6h**	26.25	26.06	26.00	32.49	32.26	32.02
**Cold-9h**	25.36	25.33	25.30	30.80	30.51	30.73
**Cold-12h**	26.59	26.46	26.24	34.48	34.10	34.02
**PEG-0h**	25.36	25.34	25.32	31.28	31.24	31.26
**PEG-1h**	26.17	26.15	26.16	31.43	31.39	31.86
**PEG-3h**	26.26	26.25	26.16	33.86	33.35	33.60
**PEG-6h**	25.09	25.15	25.07	31.23	31.21	31.22
**PEG-9h**	24.58	24.76	24.70	30.16	30.45	30.60
**PEG-12h**	26.17	26.26	26.28	30.24	30.13	30.69

Note: 6-BA, 6-Benzylaminopurine; ABA, abscisic acid; MeJA, methyl jasmonate; Cold, 4 °C; PEG, polyethylene glycol 6000.

Table S4. The quantification cycle (Cq) values of *OsRRB2* and *OsActin* in qRT-PCR analysis.

Treatments	*OsActin*			*OsRRB2*		
**6-BA-0h**	23.14	23.01	23.03	24.39	24.28	24.16
**6-BA-1h**	23.12	23.00	22.98	24.68	24.52	24.66
**6-BA-3h**	20.89	20.77	20.77	22.76	22.78	22.89
**6-BA-6h**	21.68	21.31	21.20	22.31	22.36	22.22
**6-BA-9h**	21.76	21.59	21.53	22.54	22.59	22.40
**6-BA-12h**	22.40	22.08	22.10	22.10	21.92	22.00
**MeJA-0h**	26.97	26.99	26.92	30.14	30.07	30.40
**MeJA-1h**	26.51	26.64	26.73	29.11	29.53	29.32
**MeJA-3h**	26.66	26.85	26.88	30.93	30.96	30.94
**MeJA-6h**	26.62	26.76	26.79	30.50	30.74	30.62
**MeJA-9h**	25.67	25.69	25.94	29.18	29.31	29.98
**MeJA-12h**	26.12	26.05	26.06	29.76	29.66	29.71
**ABA-0h**	27.43	27.41	27.53	27.98	27.71	27.95
**ABA-1h**	28.45	28.62	28.11	27.61	27.83	27.84
**ABA-3h**	28.50	28.48	28.36	28.57	28.70	28.54
**ABA-6h**	27.80	27.89	27.74	28.25	28.22	28.20
**ABA-9h**	29.68	29.23	29.68	29.79	29.42	29.20
**ABA-12h**	28.49	28.22	28.19	28.83	28.75	28.76
**Cold-0h**	26.32	26.26	26.26	29.79	29.96	29.49
**Cold-1h**	26.08	26.01	26.00	29.30	29.33	29.15
**Cold-3h**	26.25	26.16	26.11	29.31	29.48	29.61
**Cold-6h**	26.68	26.54	26.46	29.79	29.90	29.81
**Cold-9h**	25.90	25.93	25.84	28.34	28.55	28.69
**Cold-12h**	27.22	27.02	27.08	31.32	31.02	31.10
**PEG-0h**	25.55	25.51	25.57	29.65	29.62	29.35
**PEG-1h**	26.26	26.38	26.30	29.43	29.10	29.37
**PEG-3h**	26.26	26.31	26.24	29.54	29.70	29.89
**PEG-6h**	25.22	25.18	25.19	29.11	29.18	29.09
**PEG-9h**	24.82	24.72	24.72	28.15	28.10	28.31
**PEG-12h**	26.42	26.34	26.39	29.28	29.43	29.54

Note: 6-BA, 6-Benzylaminopurine; ABA, abscisic acid; MeJA, methyl jasmonate; Cold, 4 °C; PEG, polyethylene glycol 6000.

Table S5. The quantification cycle (Cq) values of *OsRRB3* and *OsActin* in qRT-PCR analysis.

Treatments		*OsActin*			*OsRRB3*	
**6-BA-0h**	21.20	21.21	21.07	24.53	24.55	24.12
**6-BA-1h**	21.66	21.60	21.45	25.25	25.16	25.39
**6-BA-3h**	19.60	19.46	19.43	23.01	22.97	22.97
**6-BA-6h**	20.04	19.93	19.91	22.96	22.91	22.96
**6-BA-9h**	20.38	20.13	20.15	23.19	23.15	23.20
**6-BA-12h**	20.85	20.83	20.68	22.99	23.11	22.86
**MeJA-0h**	26.71	26.63	26.44	28.21	28.28	28.35
**MeJA-1h**	26.23	26.17	26.20	27.91	27.87	27.70
**MeJA-3h**	27.00	27.00	27.08	28.07	28.61	28.25
**MeJA-6h**	27.33	27.19	27.07	27.92	28.01	28.01
**MeJA-9h**	23.69	23.74	23.70	26.13	26.48	26.30
**MeJA-12h**	25.06	25.10	25.18	26.73	26.82	26.74
**ABA-0h**	29.46	29.23	29.25	30.52	30.97	30.53
**ABA-1h**	28.83	28.90	28.81	29.26	29.38	29.48
**ABA-3h**	28.81	28.52	28.56	29.02	28.84	29.02
**ABA-6h**	28.07	28.02	28.02	28.21	28.45	28.37
**ABA-9h**	30.80	30.99	30.55	30.42	30.44	30.38
**ABA-12h**	28.58	28.41	28.63	28.92	28.95	28.88
**Cold-0h**	25.91	25.76	25.57	30.39	30.49	30.27
**Cold-1h**	24.49	24.54	24.21	30.47	30.36	30.59
**Cold-3h**	25.2	25.19	25.16	30.56	30.52	30.59
**Cold-6h**	26.26	26.03	26.03	30.5	30.61	30.4
**Cold-9h**	23.91	23.77	23.73	27.48	27.52	27.73
**Cold-12h**	26.95	26.99	26.83	31.2	31.21	31.49
**PEG-0h**	27.56	27.42	27.65	30.68	30.84	30.55
**PEG-1h**	27.55	27.30	27.37	28.38	28.71	28.06
**PEG-3h**	27.76	27.68	27.74	28.55	28.80	28.67
**PEG-6h**	24.84	24.88	24.86	31.24	31.88	31.10
**PEG-9h**	24.02	24.13	24.14	29.68	29.69	29.38
**PEG-12h**	27.11	27.19	27.15	30.48	30.50	30.25

Note: 6-BA, 6-Benzylaminopurine; ABA, abscisic acid; MeJA, methyl jasmonate; Cold, 4 °C; PEG, polyethylene glycol 6000.

Table S6. The quantification cycle (Cq) values of *OsRRB4* and *OsActin* in qRT-PCR analysis.

Treatments	*OsActin*			*OsRRB4*		
**6-BA-0h**	23.14	23.01	23.03	25.57	25.47	25.52
**6-BA-1h**	23.12	23.00	22.98	25.55	25.70	25.19
**6-BA-3h**	20.89	20.77	20.77	23.26	23.23	23.24
**6-BA-6h**	21.68	21.31	21.20	23.25	23.26	23.12
**6-BA-9h**	21.76	21.59	21.53	23.41	23.40	23.50
**6-BA-12h**	22.40	22.08	22.10	23.38	23.25	23.24
**MeJA-0h**	26.27	26.25	26.35	30.19	30.91	30.58
**MeJA-1h**	26.02	26.11	26.08	29.03	29.6	29.84
**MeJA-3h**	26.07	26.14	26.17	31.02	31.19	31.11
**MeJA-6h**	26.04	26.12	26.1	30.74	30.88	30.81
**MeJA-9h**	25.19	25.24	25.27	28.62	28.73	28.68
**MeJA-12h**	25.54	25.73	25.6	29.44	29.87	29.65
**ABA-0h**	24.00	23.98	23.81	24.62	24.48	24.53
**ABA-1h**	25.87	25.69	25.91	26.25	26.24	26.24
**ABA-3h**	26.04	26.09	26.14	26.51	26.49	26.62
**ABA-6h**	26.68	26.59	26.57	26.02	25.80	25.86
**ABA-9h**	26.91	26.99	26.69	27.64	27.62	27.64
**ABA-12h**	25.61	25.47	25.33	26.08	25.86	26.03
**Cold-0h**	25.57	25.67	25.51	30.18	30.3	30.47
**Cold-1h**	25.48	25.46	25.6	29.33	29.42	29.78
**Cold-3h**	25.63	25.65	25.62	29.11	29.2	29.08
**Cold-6h**	26.13	26.11	26.13	29.38	29.45	29.74
**Cold-9h**	25.37	25.47	25.24	28.16	28.02	28.28
**Cold-12h**	26.51	26.44	26.39	30.36	30.5	30.15
**PEG-0h**	25.40	25.38	25.40	30.12	30.30	30.21
**PEG-1h**	26.31	26.14	26.37	29.83	29.83	29.91
**PEG-3h**	26.26	26.47	26.48	29.35	29.59	29.98
**PEG-6h**	25.07	25.25	25.34	29.58	29.58	29.76
**PEG-9h**	24.87	24.61	24.85	28.59	28.75	28.98
**PEG-12h**	27.26	27.62	27.65	29.66	29.52	29.88

Note: 6-BA, 6-Benzylaminopurine; ABA, abscisic acid; MeJA, methyl jasmonate; Cold, 4 °C; PEG, polyethylene glycol 6000.

Table S7. The quantification cycle (Cq) values of *OsRRB5* and *OsActin* in qRT-PCR analysis.

Treatments	*OsActin*			*OsRRB5*		
**6-BA-0h**	23.14	23.01	23.03	26.59	26.26	26.28
**6-BA-1h**	23.12	23.00	22.98	27.31	27.13	27.15
**6-BA-3h**	20.89	20.77	20.77	25.40	25.31	25.30
**6-BA-6h**	21.68	21.31	21.20	25.78	25.67	25.75
**6-BA-9h**	21.76	21.59	21.53	25.19	25.11	25.05
**6-BA-12h**	22.40	22.08	22.10	24.93	24.73	24.92
**MeJA-0h**	28.97	28.73	28.88	32.66	32.72	32.72
**MeJA-1h**	28.44	28.39	28.50	33.27	33.72	33.26
**MeJA-3h**	29.51	29.53	29.49	33.82	33.42	33.01
**MeJA-6h**	29.87	29.86	29.65	33.54	33.51	33.22
**MeJA-9h**	26.33	26.32	26.51	31.79	31.97	31.97
**MeJA-12h**	27.55	27.71	27.66	32.88	32.62	32.93
**ABA-0h**	29.46	29.23	29.25	33.60	33.22	33.38
**ABA-1h**	28.83	28.90	28.81	32.17	31.04	31.70
**ABA-3h**	28.81	28.52	28.56	30.14	30.37	30.11
**ABA-6h**	28.07	28.02	28.02	30.75	30.01	30.46
**ABA-9h**	30.80	30.99	30.55	33.11	32.66	33.02
**ABA-12h**	28.58	28.41	28.63	31.11	30.68	30.83
**Cold-0h**	27.32	27.34	27.17	32.54	32.53	32.70
**Cold-1h**	27.08	27.02	27.06	33.57	33.10	33.17
**Cold-3h**	27.47	27.32	27.40	33.39	33.24	33.11
**Cold-6h**	28.61	28.37	28.34	33.85	33.07	33.21
**Cold-9h**	26.47	26.42	26.34	32.36	32.73	32.32
**Cold-12h**	29.59	29.36	29.10	35.75	35.96	35.94
**PEG-0h**	27.54	27.71	27.37	29.70	29.41	29.49
**PEG-1h**	27.62	27.86	27.56	30.06	30.19	30.08
**PEG-3h**	28.10	28.09	28.05	29.75	29.73	29.85
**PEG-6h**	25.05	24.97	24.91	28.51	28.67	28.66
**PEG-9h**	24.06	24.04	24.10	28.35	28.55	28.30
**PEG-12h**	27.29	27.16	27.36	30.02	29.93	29.94

Note: 6-BA, 6-Benzylaminopurine; ABA, abscisic acid; MeJA, methyl jasmonate; Cold, 4 °C; PEG, polyethylene glycol 6000.

Table S8. The quantification cycle (Cq) values of *OsRRB6* and *OsActin* in qRT-PCR analysis.

Treatments		*OsActin*			*OsRRB6*	
**6-BA-0h**	21.20	21.21	21.07	30.72	30.88	30.89
**6-BA-1h**	21.66	21.60	21.45	31.01	30.97	30.83
**6-BA-3h**	19.60	19.46	19.43	30.75	30.56	30.87
**6-BA-6h**	20.04	19.93	19.91	30.22	30.67	30.74
**6-BA-9h**	20.38	20.13	20.15	30.73	30.65	30.45
**6-BA-12h**	20.85	20.83	20.68	30.84	30.99	31.01
**MeJA-0h**	26.69	26.80	26.56	34.12	34.64	34.77
**MeJA-1h**	26.81	26.76	26.56	35.37	35.09	35.34
**MeJA-3h**	26.47	26.55	26.62	36.20	36.24	36.59
**MeJA-6h**	26.43	26.74	26.62	36.09	36.27	36.35
**MeJA-9h**	25.72	25.80	25.63	36.18	36.00	36.64
**MeJA-12h**	26.17	26.12	26.08	35.93	35.22	35.96
**ABA-0h**	27.43	27.41	27.53	31.47	31.19	31.14
**ABA-1h**	28.45	28.62	28.11	31.06	31.20	31.04
**ABA-3h**	28.50	28.48	28.36	31.07	30.92	31.13
**ABA-6h**	27.80	27.89	27.74	30.66	30.83	30.43
**ABA-9h**	29.68	29.23	29.68	30.90	30.81	30.82
**ABA-12h**	28.49	28.22	28.19	31.03	31.05	31.22
**Cold-0h**	26.82	26.5	26.49	33.03	33.27	33.13
**Cold-1h**	26.36	26.36	26.28	31.96	31.87	31.63
**Cold-3h**	29.5	29.49	29.52	32.73	32.81	32.06
**Cold-6h**	30.1	30.12	30.1	32.37	32.37	32.03
**Cold-9h**	26.26	26.15	26.13	32.12	32.59	32.06
**Cold-12h**	27.25	27.34	27.24	32.43	32.06	32.03
**PEG-0h**	25.63	25.46	25.41	33.33	33.05	33.11
**PEG-1h**	26.65	26.47	26.40	35.49	35.58	35.15
**PEG-3h**	26.52	26.58	26.46	34.06	34.12	34.59
**PEG-6h**	25.49	25.34	25.19	34.39	34.41	34.64
**PEG-9h**	24.96	24.86	24.87	34.51	34.10	34.41
**PEG-12h**	26.82	26.66	26.45	34.80	34.35	34.61

Note: 6-BA, 6-Benzylaminopurine; ABA, abscisic acid; MeJA, methyl jasmonate; Cold, 4 °C; PEG, polyethylene glycol 6000.
